# ACTION-FRANCE: Insights into Perceptions, Attitudes, and Barriers to Obesity Management in France

**DOI:** 10.3390/jcm13123519

**Published:** 2024-06-15

**Authors:** Laurence Salle, Olivier Foulatier, Muriel Coupaye, Vincent Frering, Alina Constantin, Anne-Sophie Joly, Ben Braithwaite, Fella Gharbi, Lysiane Jubin

**Affiliations:** 1 Inserm, U1094, IRD, U270, EpiMaCT—Épidémiologie des Maladies Chroniques en Zone Tropicale, 2 Rue du Dr Marcland, 87025 Limoges, France; 2 CHU de Limoges, Service d’Endocrinologie-Diabétologie-Maladies Métaboliques, 2 Avenue Martin Luther King, 87042 Limoges, France; 3 Ligue Contre l’Obésité, 24 Rue Tronchet, 75008 Paris, France; 4 Clinique Croix Saint Michel, 40 Avenue Charles de Gaulle, 82000 Montauban, France; 5 AFERO (Association Française d’Etude et de Recherche sur l’Obésité), 1 Avenue du Pr Jean Poulhès, BP 84225, 31400 Toulouse, France; 6 Centre Intégré Nord Francilien de Prise en Charge de l’Obésité (CINFO), Assistance Publique-Hôpitaux de Paris, Service des Explorations Fonctionnelles, Hôpital Louis-Mourier, 92700 Colombes, France; 7 Clinique de la Sauvegarde, Espace Médico-Chirurgical, Immeuble Trait d’Union, Entrée A29, Av des Sources, 69009 Lyon, France; 8 Collectif National des Associations d’Obèses, 62 Rue Jean Jaurès, 92800 Puteaux, France; 9 Sanoïa e-Health Services, 188 Av 2nd Division Blindée, 13420 Gémenos, France; 10 Novo Nordisk, Carré Michelet, 12 Cours Michelet, 92800 Puteaux, Francelyju@novonordisk.com (L.J.)

**Keywords:** obesity management, perception of obesity, physician–patient interaction, obesity treatment

## Abstract

**Background/Objectives:** ACTION-FRANCE (Awareness, Care, and Treatment In Obesity maNagement in France) aims to identify the perceptions, attitudes, behaviors, and potential barriers to effective obesity management in France and guide collaborative actions. **Methods:** ACTION-FRANCE is a cross-sectional survey of people with obesity (PwO) and healthcare professionals (HCPs) in France. The PwO and HCP survey questionnaire periods ran from 27 September 2022 to 1 February 2023 and from 19 December 2022 to 31 March 2023, respectively. **Results:** The study, encompassing 1226 PwO and 166 HCPs, reveals a shared recognition of obesity as a chronic condition. However, despite being requested by most PwO, weight-related discussions are surprisingly infrequent, leading to delayed diagnosis and care. PwO and HCPs held different views as to why: HCPs often attributed it to PwO’s lack of motivation or disinterest, whereas PwO avoided them because they felt weight management was their own responsibility and were uncomfortable discussing it. When weight was discussed, primarily with general practitioners (GPs), discussions mostly focused on physical activity and diet. However, results identified the strong psychosocial impact of obesity: 42% of respondents reported anxiety/depressive symptoms, and many more hesitated to engage in certain social activities because of their weight. Psychotherapy was only discussed by 55% of HCPs. Pharmaceutical options were also rarely discussed (19.5% of HCPs), though 56.1% of PwO reported they would want to. **Conclusions:** HCPs’ and PwO’s perceptions differed significantly and need to converge through enhanced communication. A holistic approach, integrating comprehensive training for GPs and recognizing psychological comorbidities, would help to bridge perceptual gaps effectively and foster more empathetic and effective patient care.

## 1. Introduction

Obesity has become a significant global public health challenge, affecting populations across the world regardless of their economic development. Obesity is characterized by an excessive accumulation of body fat, frequently leading to various health complications, and imposing significant economic burdens on society. Obesity is usually assessed in clinical practices all over the world by calculating a person’s body mass index (BMI), expressed as the ratio of body weight in kilograms to height in meters squared (kg/m^2^). According to the World Health Organization (WHO), being overweight is defined as having a BMI (body mass index) of 25 kg/m^2^ or higher, while obesity is defined as having a BMI of 30 kg/m^2^ or higher [1]. Calculating BMI is an inexpensive and easy screening method for assessing weight categories. However, BMI does not measure fat directly. Instead, it is moderately correlated with more direct measures of body fat. Furthermore, BMI appears to be as correlated with various metabolic and disease outcomes as these more direct measures of body fat [2,3,4,5,6].

France also faces the challenge of increasing obesity rates. According to recent epidemiological studies, the prevalence of excess weight in the country was 47.3% in 2020 [7]. Over the past few decades, while the prevalence of persons being overweight has fluctuated, the prevalence of obesity has steadily risen from 8.5% in 1997 to 17% in 2020 [7,8,9]. Fontbonne et al. (2023) observed that women exhibit higher obesity rates than men, increasing with age, while geographical location and profession “may” also play a role, though these results are not as clear cut [7].

Obesity places a significant burden on the affected individuals, impacting both their physical and mental well-being [10]. Excess weight and obesity pose major risks for several chronic conditions, including cardiovascular diseases such as heart disease and stroke [11], which are the main causes of death worldwide. Obesity is also associated with various other comorbidities such as diabetes [12], hypertension [13], depression [14], sleep apnea syndrome [15], and cancer [16,17], leading to a reduced quality of life and an increased risk of premature mortality. The treatment and prevention of the condition of being overweight and obesity are, therefore, essential to improve the health of the population and reduce mortality. 

Obesity is becoming ever more prevalent with the rising consumption of unhealthy foods, coupled with reduced physical activity due to changes in work patterns, greater transportation availability, and increased urbanization [1]. However, obesity extends beyond the physical and dietary aspect of the environment, encompassing social, economic, and cultural contexts. Over 100 wider factors that contribute to obesity have been identified, many of which lie beyond an individual’s influence [18]. These contributing factors include biological aspects [19,20], psychosocial influences [21], socioeconomic factors [22], and behavioral and environmental factors [23]. Thus, effectively treating obesity requires a comprehensive and collaborative approach involving healthcare professionals (HCPs), policymakers, and society. Although the condition of being overweight and obesity stem from preventable and reversible factors, no country has succeeded in stopping or curbing this epidemic yet [24]. However, some countries, including France, have succeeded in stabilizing certain categories of obesity [7].

Obesity is increasingly recognized not just as a risk factor, but as a chronic disease. However, previous studies have identified several obstacles which hinder people living with obesity (PwO) from seeking and obtaining proper medical care and support to effectively manage the condition. Obstacles include lack of awareness of the severity of obesity and the risks it poses, as well as low rates of obesity diagnosis and management [25].

Obesity management faces challenges due to stigma, discrimination, and limited familiarity with evidence-based clinical management among HCPs [26]. Obesity stigma can lead to barriers in diagnosis and treatment and negatively impact the mental health and quality of life of PwO [27]. Despite the growing obesity pandemic, there is limited research available regarding the experiences, challenges, and needs of PwO and the HCPs who treat them.

Thus, in order to overcome and meet these evolving needs, it is essential to first clearly identify and understand them. To this end, the Awareness, Care, and Treatment In Obesity maNagement International Observation study (ACTION-IO) played a crucial role in examining obesity perceptions, attitudes, behaviors, and potential barriers to management for PwO and HCPs in 11 countries across the world: Australia [28], Chile [29], Israel [30], Italy [31], Japan [32], Mexico [33], Saudi Arabia [34], South Korea [35], Spain [36], the United Arab Emirates [37], and the UK [38]. These studies, which were conducted in countries around the world from 2015 to 2018, aimed to raise awareness of the challenges faced by both groups in approaching and controlling obesity. 

The ACTION-FRANCE study adapts the structure of previous ACTION studies to specific insights in France. The objectives of the ACTION-FRANCE study were (1) to study the perceptions, attitudes, behaviors, and potential barriers to effective obesity management among people with obesity in France and (2) propose avenues to guide collaborative action to improve care, information, and support for patients with obesity.

## 2. Materials and Methods

### 2.1. Study Design and Participants

The ACTION-FRANCE study was a cross-sectional, observational, descriptive, and exploratory study that collected data via online surveys of PwO and HCPs in France. 

ACTION-FRANCE was based on the previous ACTION-IO (International Observatory) [39] and ACTION-CANADA [40] studies and was adapted to the French cultural context by a national steering committee of obesity experts. The questionnaire for the ACTION-FRANCE study was based on the previously published ACTION-IO questionnaire [39], which was translated into French. Specifically, the number of questions was reduced to avoid redundancy and increase the proportion of complete responses. Also, some elements specific to France and to the French healthcare system were added. To avoid response bias, questionnaire items were carefully phrased and presented in the same order as previous ACTION studies. 

The PwO questionnaire covered weight evolution and management, PwO’s goals and efforts, perceptions and attitudes, as well as interactions between HCPs and PwO. Themes covered by the HCP questionnaire mirrored those of the PwO questionnaire. The PwO questionnaire was divided into two parts, one mandatory and one optional. In total, it contained 58 questions (89 questions in previous studies), including 33 original questions, 20 modified questions, and 5 added questions. In addition, 22 of the original questions were optional for this study. As for the HCP questionnaire, it contains 41 original questions, 25 modified questions, and 1 added question (68 questions in previous studies). A 5-point Likert scale was used to measure attitudes or opinions. A commonly used scale within the study assessed respondents’ agreement with a statement and was measured from 1 “strongly disagree” to 5 “strongly agree”, with the midpoint of 3 as neutral. 

A third-party vendor (Sanoïa, Gémenos, France) conducted the online survey and managed the data collection and analysis. Data were collected from 27 September 2022 to 31 March 2023. Respondents were mostly recruited via online panel and via online panel companies (Kantar France, Paris, France) and patient organizations (PwO only). All patients had previously given permission to be contacted for research purposes.

Eligible PwO were aged 18 years or older, with a current body mass index (BMI), based on self-reported height and weight, of at least 27 kg/m^2^ and residing in France (mainland and overseas territories). Exclusion criteria were previous participation in this study (i.e., having previously given online consent for this study), pregnancy, mental incapacity, and language barriers precluding adequate understanding or cooperation. Ten PwO whose height was less than 120 cm were excluded from the analysis population as data management processes identified major inconsistencies in data provided for this population.

Eligible HCPs were any healthcare professional involved in the management of obesity (doctor, nurse, surgeon, pharmacist, dietician, psychologist, medical assistant, physiotherapist, or midwife), residing and practicing in France (mainland and overseas territories), aged over 18. Note that ACTION-FRANCE has accepted bariatric surgeons, unlike previous ACTION-IO studies. Also, although being pregnant was an exclusion criterion, midwives were included, given that in France they are involved in the care of non-pregnant patients, notably by prescribing contraception. For ethical and legal reasons, no distinction was made in terms of ethnic and racial affiliation.

Screening questions were used to determine eligibility based on these demographic targets. Respondents who passed the screening process, had a BMI of at least 27 kg/m^2^, and who met the other study eligibility criteria, were permitted to complete the full survey (Table S1).

### 2.2. Statistical Analysis

Responses were considered complete when all mandatory questions were answered. Only complete responses were analyzed using descriptive statistics according to the obesity class (based on self-reported weight and height) and the current success (having generally lost weight over the past year) or non-success (having regained weight in the past year or having maintained a stable weight in the past year) of weight loss and treatment-seeking behavior (Table S1). The obesity classes were determined as follows: being overweight, with a body mass index (BMI) of 27–29.9 kg/m²; Class I obesity, with a BMI of 30–34.9 kg/m²; Class II obesity, with a BMI of 35–39.9 kg/m²; and Class III obesity, with a BMI of ≥40 kg/m² [41].

For qualitative data, the frequency and percentage for each modality were calculated. Numerical data were presented as median (and IQR) or mean (and SD), as specified in the text. 

All calculations were made with SAS for Windows (v 9.4; SAS Institute Inc., Cary, NC, USA) and R software (v 4.2.3; R Foundation for Statistical Computing) was used for graphs.

## 3. Results

### 3.1. Participants

In total, 1493 PwO answered the questionnaire. Based on the screening questions, 267 PwO were excluded from the analysis, leading to an analysis population of 1226 PwO (Figure S1A). Additional questions were answered by 1108 PwO. Regarding HCPs, 185 participants answered the questionnaire, of which 19 were excluded. The analyzed population of HCP consisted of 166 respondents (Figure S1B).

The mean age of PwO (n = 1226) was 48.4 ± 13.6 years, and respondents ranged between 18 and 83 years of age. The majority of respondents (74.1%) were female (Table 1).

Concerning PwO’s medical characteristics, 14.9%, 43.1%, 22.5%, and 19.9% of the respondents belonged to the following BMI class: BMI < 30 kg/m², Class I, Class II, and Class III, respectively. Bariatric surgery was performed for 11.4% of the respondents. The mean number of comorbidities was 2.2 ± 1.9 (Table 1), with the most frequent being depression/anxiety (42.0%), hypertension (38.7%), and obstructive sleep apnea (35.3%) (Table S2). In addition, 190 PwO achieved weight loss (15.5%) and 433 were actively seeking treatment (35.7%). 

Considering the HCP population (n = 166), 12.7% were dietitians (n = 21) and 64.5% were doctors (n = 107), of whom 48.7% were general practitioners, 21.5% nutrition specialists, 10.3% endocrinologists, and 8.4% bariatric or obesity surgeons. Among the rest of the HCP population, which accounted for 22.9% (n = 38), various professions were represented, including 12.7% pharmacists (n = 21), 6.6% nurses (n = 11), 2.4% psychologists (n = 4), and 1.2% physiotherapists (n = 2). Obesity specialists (i.e., a physician for whom ≥50% of adult patients are seen primarily for weight management) represented 65.7% of the HCP population. The mean time in practice was 19.5 ± 10.6 years. Each HCP was in charge of around 20 patients (median and IQR: 6.0–43.8) specifically dedicated to obesity management during the last month prior to completing the questionnaire (Table 1).

### 3.2. Perception of Obesity

**Figure 1:**
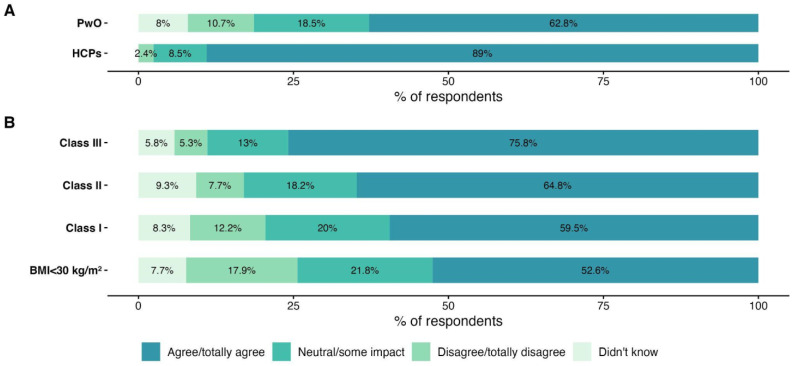
Perception of obesity as a chronic disease by HCPs and PwO (**A**) and according to BMI class, for PwO only (**B**).

Among PwO, 62.8% (n = 678) believed that obesity is a chronic disease, which was a significantly lower percentage than for HCPs, among whom 89% held the same view (Figure 1A). The perception of obesity as a chronic disease seemed to be correlated with BMI class, with a higher prevalence of this belief observed as BMI increased (Figure 1B). Compared to other diseases, including diabetes, chronic obstructive pulmonary disease, stroke, and cancer, obesity was perceived by HCPs as having a lesser impact on health, while PwO considered it to be their most significant health issue (Figure S2A). However, 76.5% of HCPs considered obesity to be at least as important as the other diseases they treat. 

When asked about life expectancy, 82.8% of PwO felt that being overweight or obesity would reduce it. According to HCPs, life expectancy decreases with BMI, from a minimum of 3.8 ± 6.7 years to a maximum of 13.5 ± 8.5 years for overweight and Class III individuals, respectively (mean for each class), and with a mean loss reduction of 10.2 ± 7.8 years for all obesity classes.

PwO tended to underestimate their own weight status, and this was especially noticeable among respondents with medical obesity as 47.3% of them considered themselves overweight and not in a situation of obesity. Similarly, 46.9% of PwO with Class III obesity thought of themselves as having obesity but not extremely severe obesity. Interestingly, a significant majority (74.9%) of people with a BMI < 30 accurately perceived their weight category (Figure S2B).

### 3.3. Psychological and Social Impact of Obesity

**Figure 2:**
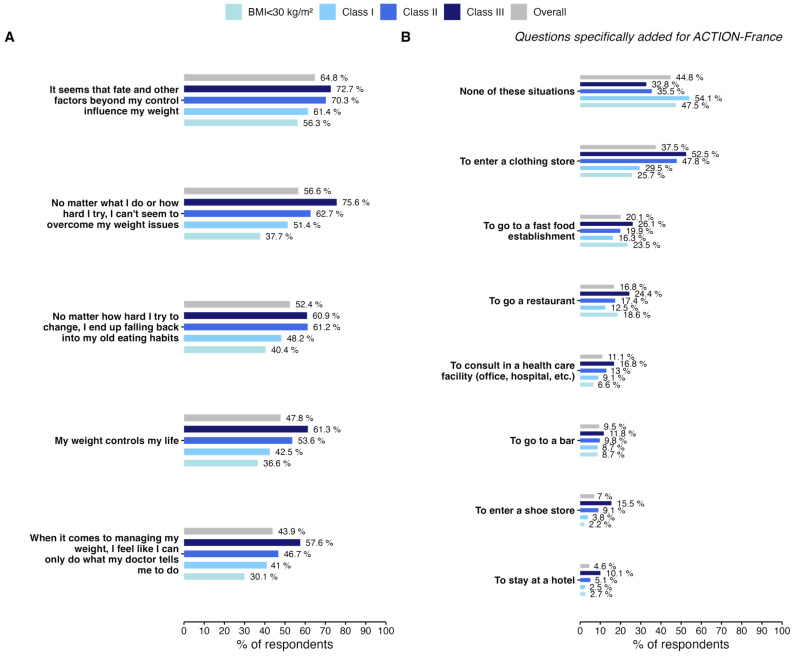
Psychological and social impact of obesity by BMI class. (**A**) Psychological dimensions of obesity. (**B**) Impact on social activities. Questions on the impact of obesity on social activity were specifically added to the ACTION-FRANCE questionnaire. For both plots, percentages correspond to the percentage of PwO who answered “agree” or “totally agree”.

PwO reported a feeling of powerlessness concerning their weight. The prevalence of this sentiment appeared to increase with BMI (Figure 2A). For instance, 47.8% of PwO expressed the belief that their weight controls their lives, increasing to 63.1% among those in BMI Class III. Additionally, 56.6% of PwO said they were unable to lose weight, no matter how hard they tried, reaching 75.6% for those in Class III. Furthermore, 64.8% of PwO responding believed that their weight was controlled by fate or other uncontrollable factors, and this rose to 72.7% for PwO belonging to BMI Class III.

Regarding social activities, over half of the respondents (55.2%) expressed feelings of concern about the situations presented, and this trend is particularly pronounced among people with a BMI over 34.9 (Figure 2B). Half of the PwO belonging to BMI Classes II and III have already hesitated or refrained from going to a clothing store due to their weight (47.8% and 52.5%, respectively). Approximately one in four respondents in BMI Class III and those with a BMI < 30 have previously decided not to enter a fast-food restaurant. Alarmingly, approximately 1 out of every 10 respondents hesitated or decided against the idea of seeking medical care in a healthcare facility.

### 3.4. Weight-Loss Goals, Success, and Motivations

The most important weight management goals for PwO included losing any amount of weight (56.5%), improving their appearance (36.3%), preventing health problems (33.6%), and gaining more energy (26.6%), as well as increasing their lifespan (23.9%). In terms of the amount of weight to lose, 87.3% of PwO believed that a 5–10% reduction in body weight would be extremely beneficial for their health. However, PwO’s self-reported ideal weight was 24.0 ± 13.1% (n = 1096) lower than their current weight.

The goals most frequently set with healthcare professionals were to lose any amount of weight (44.1%), stop gaining weight (28.3%), reduce weight-related risks (21.7%), improve existing health conditions (21.5%), and improve physical and mental well-being (21.5%).

Indeed, HCPs prioritized improving the existing health status (55.4%), enhancing both physical and mental well-being (49.4%), and promoting an overall improvement in lifestyle (48.8%), whereas a goal of improving appearance is only set 16.9% of the time.

It is noteworthy that 17.7% of PwO had no specific weight management goals. Yet a majority of PwO (58.8%) and HCPs (74.7%) believed that setting realistic and attainable goals significantly improves their chances of success in weight loss or management endeavors. The other most frequent contributors to weight loss success were support from family, colleagues, or friends (46.3% and 55.4%) and from HCPs (42.3% and 54.8%), along with personal motivation and determination to lose weight (34.5% and 31.3%) for both PwO and HCPs, respectively.

According to HCPs, the greatest motivator for patients to lose weight was their overall health status, as this was reported by 79.5% of HCPs, followed by specific personal medical events and diagnoses (72.3%), their desire to improve their physical well-being and energy (63.9%), and for improved self-esteem (51.8%). PwO’s reported motivators were similar, though PwO most often prioritized reaching a weight where they feel comfortable (61%), with overall health status a far second (46.1%). Other common motivators included the desire to improve their physical well-being and energy (42.6%), to fit into smaller-sized clothing (34.2%), and for improved self-esteem (29.4%). Interestingly, 14.5% of HCPs reported improved sex life as a motivator, while this frequency was lower for PwO, at 7.8%.

### 3.5. Previous Weight Loss Attempts: Outcomes and Barriers

About 1 in 10 (8.8%) PwO reported having generally lost weight in the last 10 years, while 68.9% said they had made at least one attempt to lose weight, with an average of six attempts per respondent during their adult life. Some 46.3% of PwO have lost at least 5% of their body weight in the last 3 years.

**Figure 3:**
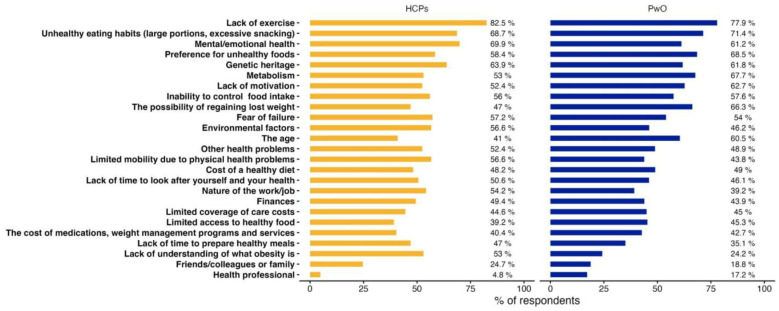
Barriers to weight loss reported by PwO and HCPs. Percentages correspond to the percentage of PwO who answered “agree” or “totally agree”.

Regarding barriers to weight-loss, HCPs and PwO hold generally similar views (Figure 3). The primary barriers reported by both groups include lack of exercise (82.5% and 77.9%, respectively), unhealthy eating habits (68.7% and 71.4%), mental and emotional health (69.9% and 61.2%), and genetic heritage (63.9% and 61.8%). Both populations agreed that friends, colleagues, or family, as well as health professionals, did not constitute significant barriers. Additionally, almost half of all respondents (48.2% and 49%) pointed out that the cost of healthy food serves as a hindrance to weight loss.

However, there were notable differences in certain aspects. For instance, 53% of HCPs considered the lack of understanding of obesity to be a significant barrier, while only 24.2% of PwO shared this view. Additionally, 41% of HCPs mentioned age as a potential barrier, while 60.5% of PwO considered it relevant. PwO identified the lack of motivation (62.7%), the fear of regaining lost weight (66.3%), and the nature of a PwO’s job (54.2%) as significant barriers, compared to 52.4%, 47%, and 39.2% of HCPs, respectively.

### 3.6. Appointments and Conversations between PwO and HCPs

In total, 816 PwO (67.5%) reported having discussed their weight or weight loss with a HCP in the last 5 years. Of these, 87.1% had discussed being overweight and 76.2% weight loss, and 17.5% reported having had a follow-up appointment or call about their weight after their last visit. Both PwO and HCPs reported initiating discussions about weight approximately half of the time. Patients most often discussed these topics with general practitioners (GPs) (69.5%).

The average age at which participants consulted a HCP about their weight was 30.4 ± 15.5 years (n = 708/1226), whereas they reported having become aware of being overweight at an average age of 28.4 ± 14.4 years (n = 1044/1226). Among PwO, 51.1% received an official diagnosis of obesity from qualified HCPs. Considering only respondents who provided responses to questions regarding age, weight, and consultations, 56.2% (n = 245/436) consulted for the first time about their weight after their BMI exceeded 29, with a mean delay of 8.7 ± 7.9 years. 

**Figure 4:**
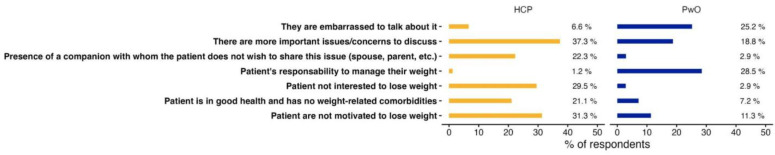
Reasons for not discussing weight management or obesity. Only reasons where a difference of at least 10% between HCPs and PwO was observed are presented. Not presented: 13.7% of PwO selected “no reason” and 12.0% of HCPs selected “other”.

Important divergences exist between the points of view of HCPs and PwO in terms of why patients would not discuss their weight with a HCP. For instance, HCPs more readily attribute this to patients’ lack of motivation (31.3%) or their disinterest in losing weight (29.5%). However, the vast majority of PwO are interested and motivated to lose weight (Figure 4). HPCs also underlined that there are other more significant issues to discuss with patients (37.3%). On the other hand, PwO refrain from initiating discussions mostly because they consider that it is entirely their own responsibility to manage their weight (28.5%) or because they do not feel comfortable discussing weight matters with their HCP (25.2%) (Figure 4). Interestingly, 15.6% of HCPs were also not comfortable initiating a conversation about their patient’s weight unless the patient mentioned it first. However, 60.2% of PwO (n = 462/1226) like it when their HPC brings up the subject of their weight during consultations. In addition, 79.3% of PwO who usually start a conversation about their weight would like to discuss it during the consultation.

### 3.7. Attitude towards Obesity Management

PwO and HCPs had divergent views regarding who was responsible for treating obesity. Indeed, PwO reported getting information on obesity and obesity management more often from internet research (40.2%) than from doctors and dietitians (36% and 29.7%, respectively). 

**Figure 5:**
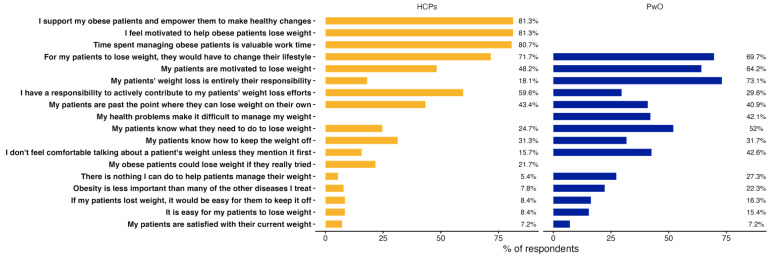
Attitudes toward obesity, as reported by HCPs and PwO. Percentages correspond to the percentage of respondents who answered “agree” or “totally agree”. Item wording is presented for HCPs; wording was modified for the PwO questionnaire (e.g., “My patients’” becomes “My” or “I”).

When asked, 73.1% of PwO agreed that weight management was entirely the patient’s responsibility, compared to only 18.1% of HCPs (Figure 5), and only 29.6% of PwO believed their HCP has a responsibility to actively contribute to their patients’ weight loss efforts, compared to 59.6% among HCPs. Perceptions of PwO’s knowledge also differed, with 52% of PwO reporting they know how to manage their weight, whereas only 24% of HCPs believed this to be the case for their patients.

When asked to choose the actors responsible for obesity treatment, both PwO and HCPs selected healthcare professionals most frequently (72.5% and 90.1%, respectively). The same was observed for obesity prevention (72.4% and 80.7%). 

HCPs had a mostly positive and encouraging attitude towards obesity management, with a large majority of HCPs agreeing that the time devoted to managing patients with weight problems is valuable work time (80.7%), and reporting that they support and empower them to make healthy changes (81.3%) as well as being motivated to help them lose weight (81.3%) (Figure 5).

### 3.8. Perception of Treatments for Obesity

**Figure 6:**
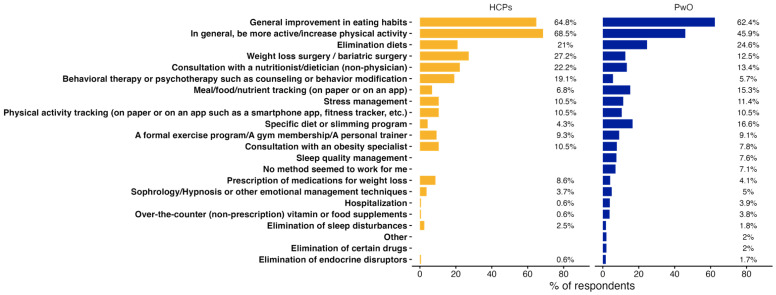
Effective weight loss methods, as reported by HCPs and PwO respondents. Percentages correspond to the percentage of PwO or HPCs who answered “Yes” to the proposition.

Physical activity was often recognized as one of the most effective methods for weight loss (PwO = 45.9% and HCPs = 68.5%), as was improving dietary habits (PwO = 62.4% and HCPs = 64.8%) (Figure 6). Stress management is considered effective by 11.4% of PwO, which is nearly the same percentage as for bariatric surgery (12.5%). Interestingly, 79.1% of healthcare professionals recommended stress management often or systematically, while only 10.5% considered it to be effective. In comparison, while bariatric surgery is more frequently evaluated as an effective method (27.2%) by HCPs, only 26.9% recommend it often or systematically. Of these 27.2% of HCPs, 22.7% were nutrition specialists/medical nutritionists (n = 10/44) and 15.9% were either endocrinologists or GPs (n = 7/44). All surgeons (n = 2/2), 85.7% of diabetes educators (n = 6/7), 66.7% of bariatric surgeons/obesity surgeons (n = 6/9), and 63.6% of endocrinologists (n = 7/11) considered bariatric surgery as an effective method. Psychotherapy is acknowledged as effective by 19.1% of HCPs, like a consultation with a nutritionist/dietitian (22.2%). However, 45% of HCPs state that they do not frequently discuss these options. 

Patients’ attitudes towards the surgical treatment of obesity are quite nuanced. Although 63.1% of patients felt well-informed about the risks and failures associated with surgery, fewer than one-third (33.8%) believed that there are currently excellent surgical solutions for weight loss and only 28.9% of patients believed that surgery is more effective than other available treatments. The cost of these procedures is perceived as a major obstacle by 35.2% of patients. Furthermore, 71.9% of participants considered that undergoing weight loss surgery would entail a permanent lifestyle change. Finally, 68.5% of patients acknowledge that weight can return after a weight loss procedure.

HCPs determined their recommendations based on their effectiveness. HCPs tended to use multiple strategies to assess the effectiveness of weight management, with 68.1% of HCPs using BMI reduction, 54.2% using improvements to patient quality of life, and 51.8% using patient satisfaction, among others.

Pharmaceutical weight loss treatments are not frequently discussed during consultations, with only 9.5% of PwO mentioning discussions about it, and 18.5% of HCPs recommending them often or systematically. Indeed, nearly half of HCPs (49.3%) reported that weight loss medications are no more effective than other weight loss treatment options. Nevertheless, 56.1% of PwO would like their HCP to propose a prescription to aid in weight loss, even though 53.4% of PwO would prefer to lose weight on their own rather than relying on medications and most PwO expressed concerns about potential short- and long-term side effects (60.2% and 58.6%). When asked whether there are good options when it comes to prescribed medications for weight loss, 26.9% of PwO did not answer and 31.3% had a neutral opinion. 

In addition, 35.7% of HCPs (n = 54/151) reported they felt they did not know enough about prescription weight loss drugs to feel comfortable prescribing them. Among these 54 HCPs, 29 (53.7%) were physicians (16 [29.6%] GPs, 6 [11.1%] nutrition specialists/medical nutritionists, and 7 [13.0%] physicians of other specialties). Among GPs specifically, 30.8% (n = 16/52) felt they did not know enough about prescription weight loss drugs. This proportion was higher among nurses (63.6%, n = 7/11) and dieticians (42.9%, n = 9/21) and lower among pharmacists (23.8%, n = 5/21). Approximately 34% of HCPs indicated that there are currently good options for prescribing weight loss medications and 61.4% cited cost as a significant barrier to prescribing such medications.

### 3.9. Ideas for Change 

The French social security system and society fail to meet the requirements of PwO according to 51.9% of HCPs and 49.2% of PwO. Among factors that could improve obesity management, HCPs selected “increasing the number of health professionals managing obesity as a chronic disease” (52.4%) most often. Several other propositions, such as increasing the number of people who view obesity as a medical condition (39.2%), providing solutions to help healthcare professionals treat patients (38.6%), changing public perceptions of people with obesity (36.8%), or the judgments made by some health professionals (36.1%), were also considered as good options for improvement. 

## 4. Discussion

The ACTION-FRANCE study highlighted several critical gaps in obesity management, most notably between the perceptions and attitudes of PwO and those of HCPs. 

Firstly, in terms of awareness, approximately two-thirds of French PwO and 90% of French HCPs viewed obesity as a chronic disease, which is in line with the situation in other countries [39]. Indeed, obesity is widely recognized by both PwO and HCPs as a serious chronic disease. Although a smaller proportion of PwO viewed obesity as a chronic disease, they considered obesity to be the factor that has the greatest impact on their health. PwO still consider obesity to be their most significant health issue; this observation lends further credence to the existing literature [42]. Therefore, there appears to be a discrepancy between PwO’s views of themselves and of their health status.

Though HCPs view obesity as having a lesser impact compared to other diseases, they, nevertheless, recognize that obesity is a serious condition, and its management should be taken seriously. Indeed, a majority of HCPs tended to overestimate the reduction in life expectancy. According to the OECD, obesity and its associated health issues reduce life expectancy by 0.9 to 4.2 years [43], which is two to three times lower than the estimates provided by HCPs. These data cast a somewhat negative light on the situation. 

Individuals with a BMI < 30 were more likely to have an accurate perception of their weight status, while a considerable number of PwO (BMI ≥ 30) tended to underestimate their BMI class. 

In addition, one of the specific features of the ACTION-FRANCE study was to ask PwO about pleasant social activities, such as going to a bar, or a restaurant, staying at a hotel, or shopping, which are social activities specifically linked to French culture. Over half of all respondents have already given up or hesitated to carry out one of these activities. Troublingly, the fact that more than 1 in 10 PwO has hesitated to consult a healthcare facility seems to indicate that progress remains to be made in encouraging treatment-seeking behavior as further evidenced by the low number of patients actively seeking treatment (35.3%). 

The reluctance of PwO to participate in certain activities also aligns with the prevalence of major comorbidities, with around one out of every three PwO exhibiting symptoms of anxiety and depression. Indeed, the impact of obesity extends beyond hypertension, diabetes, hypercholesterolemia, and cardiovascular disease. Obesity affects the well-being of individuals, with anxiety and depression being the primary comorbidities, especially in those with severe obesity. However, observed prevalence was higher than the 2020 Obepi-Roche Study [7], potentially due to the COVID-19 pandemic, which has tended to bring psychological problems such as anxiety and depression to the forefront of people’s minds and led to a surge in the prevalence of depressive symptoms [44]. To the authors’ knowledge, the association between anxiety/depression and obesity was not always emphasized in other ACTION studies [31,36], but it has been widely reported in the literature [45]. Anxiety and depression are rarely reported in ACTION studies, except in Chile, where a prevalence of 39% was reported [29], which is close to what was found in this study (42%). As a result, anxiety and depression have received little attention as obstacles to weight loss, even though mental health problems are recognized as major issues in the treatment of obesity.

Secondly, concerning healthcare, results indicated a need to enhance communication between PwO and HCPs. This finding is consistent with studies in other countries [31,40]. Most PwO reported having discussed their weight or weight loss with a HCP in the last 5 years, most often discussing these topics with GPs, highlighting the pivotal role of the GP in obesity management. Nevertheless, the majority of PwO wait for approximately ten years after becoming aware of their excess weight before seeking medical advice.

Both PwO and HCPs agree that clear and attainable goals are the most important contributor to weight loss success. Losing any amount of weight was reported as the main option goals for both populations, while improving appearance was a more important goal for PwO than for HCPs. Also, the absence of a specific weight target may correspond to a population that is more concerned with resolving comorbidities or improving their quality of life. The strictly weight-based targets are a simple but very restrictive approach to treating this condition.

In addition, weight or obesity is not always discussed during medical appointments, despite patients expressing a desire for it. They would like HCPs to initiate these discussions. HCPs should be less reluctant to broach the subject of weight with their patients during consultations since a majority of patients want to discuss it. The perceived reasons for this constitute another glaring difference between HCPs and PwO. HCPs attribute patients’ reluctance to discuss weight to a lack of motivation or a disinterest in losing weight whereas PwO avoid the discussion because they believe it is their responsibility to manage it on their own and feel uncomfortable talking about it with HCPs. The notion of responsibility for weight loss is in line with the findings from other countries, as evaluated in ACTION-IO, US ACTION, or ACTION-CANADA, where three-quarters or more of PwO surveyed held this view [39,40]. 

Patients may appear less motivated by weight loss not due to disinterest but rather because of their concern for associated comorbidities or a holistic perspective on their health that extends beyond mere consideration of the number on the scale, however, this remains to be verified. These differences highlight the importance of an open and empathetic communication to effectively address weight-related issues. Communication about weight between PwO and HCPs could be strengthened by taking steps to relieve some of the PwO’s feelings of responsibility for managing their weight. In fact, all international ACTION studies, including France, found the main reason patients did not initiate a conversation about weight management with a healthcare professional was that they felt entirely responsible for managing their weight. 

Thirdly, 1 in 10 PwO reported having lost weight in the last 10 years, while a large majority made an effort and attempted to lose weight, suggesting that management options proposed by HCPs are not effective or not adapted to patients’ needs. Limited access to specialized obesity consultations often results in a lack of awareness and support for effective and appropriate weight loss methods. Therefore, it is crucial to train healthcare professionals and all those involved to improve early detection and effective management. An important figure to bear in mind is that only half the people surveyed had received a clinical diagnosis for their obesity.

While the psychological impact of obesity, particularly the feeling of helplessness, emerges as a central element of a PwO’s life with the disease, the management options proposed by the primary healthcare provider (general practitioner), and accepted by the PwO, remain primarily focused on diet and physical activity. This approach is likely influenced by the guidelines provided by the French National Nutrition Health Program (*Plan National Nutrition Santé*, PNNS) [46], which aims to improve the overall health of the French population. This concept is widely accepted internationally, but our results suggest that French HCPs put more of an emphasis on physical activity compared to dietary changes (68.5% vs. 64.8%). This inversion is discreet but disrupts the order of the French flagship slogan “*Manger Bouger*” (“Eat Move”) to “*Bouger Manger*” (“Move Eat”).

The importance of stress management and psychotherapy are the main differences in the perception and management of obesity between PwO and HCPs. Insufficient consideration being given to managing anxiety, depression, and stress by HCPs could lead to a negatively impacted overall quality of life, as experienced by PwO, as these conditions remain untreated. Mental and emotional health problems are considered one of the main obstacles to weight loss. An adaptable treatment approach is necessary to address these differences effectively. Empowerment of patients with the tools and knowledge to actively participate in their own well-being can greatly enhance their ability to cope with the challenges of obesity and its associated mental health issues. This, in turn, should lead to adopting a new flagship slogan “*S’estimer, bouger, manger*” (“Esteem, move, eat”). 

Considering medication prescribed to lose weight, results suggest that there is a strong demand among PwO for prescription weight loss medication in France, with over half of all respondents saying they would like to discuss this option with their HCP. This is in line with the results of the ACTION-IO study. Reassuringly, expectations were realistic for the most part, and PwO appeared to be cautious regarding potential short- and long-term side effects, suggesting a healthy level of skepticism among French PwO. This enthusiastic yet markedly cautious outlook on new pharmacological treatments might change as the arrival on the market of new therapies, backed by promising results in terms of clinical effectiveness, may tend to temper this particularly cautious perception [47].

Among other weight loss methods, bariatric surgery was not perceived as an effective treatment. Indeed, only 11.4% of respondents underwent surgery. This may be a factor in the responses among PwO. This can also be explained by the fact that the data collection method (patient associations and social networks) is likely to introduce bias among respondents by recruiting more patients for whom surgery has failed to yield the expected outcomes, or who are in search of new therapeutic solutions, than patients who have had a very satisfactory outcome. 

It should be emphasized that the largest number of responders were GPs (49%), who are less trained in surgery than dedicated surgeons. This was also the case for ACTION-IO (51%). However, unlike ACTION-IO studies [39], bariatric and plastic surgeons were included in the HCP analysis population. This choice was made in view of the particularly high rate of bariatric surgery in France compared with other countries [48]. Also, the difficulties of long-term follow-up, with a high number of post-operative PwO lost to follow-up, may negatively alter the perception of this option. The study highlighted the importance of providing comprehensive information to PwO and to improve HCP’s knowledge about bariatric surgery, its costs, and long-term implications. Social networks, the primary source of information used by patients, could serve as a lever for changing perceptions of obesity treatments.

The majority of the time, PwO educate themselves about their weight and weight loss techniques, primarily relying on the internet and social media. The content of these sources likely accounts for some of the disparities in perceptions between PwO and HCPs. It would be valuable to investigate this aspect further and identify the key actors and the content of these emerging sources.

HCPs view themselves as the primary actors in obesity prevention, placing themselves well ahead of social security, government, and political decision-makers, as well as the food industry. However, physicians are not the sole contributors to tackling obesity. For example, pharmacists, nurses, and school health professionals are also key players [49]. Furthermore, it is crucial not to overlook the impact of industrialization and the accountability of public authorities in tackling obesity. Addressing food regulation becomes a key aspect to be considered, aligning with WHO recommendations to combat this pandemic. 

Finally, this study presents some limitations. Firstly, unlike other surveys, ACTION-FRANCE was conducted between 27 September 2022 and 31 March 2023. The survey’s timing raises concerns about comparisons due to the addition of three years of global pandemic history, including two years affected by the COVID-19 epidemic, which undoubtedly altered the perception of obesity both for the public and for PwO, even if it is challenging to quantify. Additionally, the questionnaires were complex, long, and dealt with several themes, potentially dissuading respondents from completing them. A more concise format with a specific theme could gain in popularity with respondents.

Also, the PwO population was predominantly female, with nearly three out of four respondents being women. This results in an overrepresentation of this group in the survey, as the prevalence of obesity, while slightly higher for women, remains similar at 16.7% for men and 17.4% for women [7].

Another limit is the geographical distribution of respondents, where regions heavily involved in obesity management presented a low response rate. Similarly, the most actively involved HCPs in the discussions are GPs, accounting for nearly 70% of respondents, yet they constitute only 45% of the overall population. Although they are the majority, this discrepancy may contribute somewhat to the observed gaps between the PwO and HCP results.

Moreover, the self-reported weight and height data for calculating BMI introduce a probable bias, resulting in an underestimation of BMI. Indeed, similarly to respondents’ skewed perception of their weight class, people will tend to understate their weight while overstating their height. Thus, while BMI is widely used, it has its limitations, as it does not consider factors such as muscle mass or body fat distribution. Therefore, in some instances, other measures, such as waist circumference and waist-to-hip ratio, may be used to further assess an individual’s risk of obesity-related complications [2]. However, implementing these measures in this study would not have addressed any potential biases generated by the declarative nature of data collection.

## 5. Conclusions

In conclusion, HCPs’ and PwO’s perceptions differ significantly. To achieve better outcomes, it is vital to break down communication barriers and dispel false beliefs, especially those held by HCPs towards PwO. Comprehensive training of HCPs, particularly general practitioners, would be useful to ensure the widespread adoption of a holistic management approach that goes beyond simple recommendations on diet and physical activity. Additionally, addressing psychological comorbidities and broadening the therapeutic arsenal with additional medical and pharmaceutical options are essential steps in combating obesity effectively. 

The study also highlights the utmost importance of recognizing obesity as a chronic disease. Increasing the number of healthcare professionals specializing in managing obesity as a chronic disease and providing solutions to help address the judgments made by some HCPs constitute the first steps towards improving obesity management. By addressing these aspects collectively, we can pave the way towards a healthier future for individuals affected by obesity and tackle the growing obesity pandemic. 

## Figures and Tables

**Table 1:** Sociodemographic and clinical description of the two populations surveyed: PwO and HCPs.

	PwO	HCPs
Characteristic	N = 1226 ^1^	N = 166 ^1^
Age (years)		
Median (IQR)	48.5 (38.0–59.0)	47.0 (38.0–57.8)
Mean (SD)	48.4 (13.6)	47.9 (11.2)
Range	18.0–83.0	26.0–73.0
Sex		
Female	903 (73.7%)	93 (56.0%)
Male	317 (25.9%)	73 (44.0%)
Other	6 (0.5%)	
Obesity class		
Class I	529 (43.1%)	8 (5.2%)
Class II	276 (22.5%)	1 (0.6%)
Class III	238 (19.4%)	
BMI < 30 kg/m²	183 (14.9%)	145 (94.2%)
Unknown		12
Bariatric surgery	140 (11.4%)	
Current weight loss		
Current weight loss Non-Success	1036 (84.5%)	
Current weight loss Success	190 (15.5%)	
Seeking treatment		
Non-treatment seeking	793 (64.7%)	
Actively seeking treatment	433 (35.3%)	
Number of comorbidities		
Median (IQR)	2.0 (1.0–3.0)	
Mean (SD)	2.2 (1.9)	
Range	0.0–18.0	
Medical specialty		
General practitioner		52 (48.6%)
Nutrition Specialist/Medical Nutritionist		23 (21.5%)
Endocrinologist		11 (10.3%)
Bariatric Surgeon/Obesity Surgeon		9 (8.4%)
Diabetes Educator		7 (6.5%)
Surgeon		2 (1.9%)
Other		1 (0.9%)
Rheumatologist		1 (0.9%)
Specialist in Internal Medicine		1 (0.9%)
Unknown		59
Obesity specialist		109 (65.7%)
Practice time (years)		
Median (IQR)		19.0 (11.0–28.8)
Mean (SD)		19.5 (10.6)
Range		1.0–43.0
Number of patients specifically dedicated to obesity management		
Median (IQR)		20.0 (6.0–43.8)
Mean (SD)		34.8 (42.7)
Range		0.0–250.0
Profession		
Doctor		107 (64.5%)
Dietitian		21 (12.7%)
Pharmacist		21 (12.7%)
Nurse		11 (6.6%)
Psychologist		4 (2.4%)
Physiotherapist		2 (1.2%)

^1^ n (%).

## Data Availability

The data are not publicly available due to legal reasons related to data privacy.

## Supplementary Materials

The following supporting information can be downloaded at https://www.mdpi.com/article/10.3390/jcm13123519/s1. Figure S1: Flow chart of the ACTION-FRANCE study for PwO (A) and HCPs (B) and Figure S2: Perception of obesity and impact of obesity on general health status by HCPs and PwO. (A) Response to the question ”What is the influence of each item on an individual’s health”, according to both populations. The percentage represents the proportion of responses for each Likert scale item. (B) PwO’s perception of their weight in relation to their clinical weight. Clinical weight was defined as follows: overweight (BMI ≤ 29.9), obesity (BMI ≥ 30 and BMI ≤ 39.9, Class I and II), and extreme obesity (BMI ≥ 40, Class III). The percentage corresponds to the total number of respondents by clinical category; Table S1: Stratifications of respondents and Table S2: PwO’s comorbidities according to BMI class.

## References

[1] World Health Organization (2022). WHO European Regional Obesity Report 2022.

[2] Khan I., Chong M., Le A., Mohammadi-Shemirani P., Morton R., Brinza C., Kiflen M., Narula S., Akhabir L., Mao S. (2023). Surrogate Adiposity Markers and Mortality. JAMA Netw. Open.

[3] Flegal K.M., Graubard B.I. (2009). Estimates of Excess Deaths Associated with Body Mass Index and Other Anthropometric Variables123. Am. J. Clin. Nutr..

[4] Freedman D.S., Katzmarzyk P.T., Dietz W.H., Srinivasan S.R., Berenson G.S. (2009). Relation of Body Mass Index and Skinfold Thicknesses to Cardiovascular Disease Risk Factors in Children: The Bogalusa Heart Study. Am. J. Clin. Nutr..

[5] Willett K., Jiang R., Lenart E., Spiegelman D., Willett W. (2006). Comparison of Bioelectrical Impedance and BMI in Predicting Obesity-Related Medical Conditions. Obesity.

[6] Sun Q., van Dam R.M., Spiegelman D., Heymsfield S.B., Willett W.C., Hu F.B. (2010). Comparison of Dual-Energy x-Ray Absorptiometric and Anthropometric Measures of Adiposity in Relation to Adiposity-Related Biologic Factors. Am. J. Epidemiol..

[7] Fontbonne A., Currie A., Tounian P., Picot M.-C., Foulatier O., Nedelcu M., Nocca D. (2023). Prevalence of Overweight and Obesity in France: The 2020 Obepi-Roche Study by the “Ligue Contre l’Obésité”. J. Clin. Med..

[8] Charles M.-A., Eschwège E., Basdevant A. (2008). Monitoring the Obesity Epidemic in France: The Obepi Surveys 1997–2006. Obesity.

[9] Inserm; Kantar Health; Roche Enquête Épidémiologique Nationale Sur Le Surpoids et l’obésité 2012. https://presse.inserm.fr/wp-content/uploads/2012/10/obepi_2012.pdf.

[10] Sarwer D.B., Polonsky H.M. (2016). The Psychosocial Burden of Obesity. Endocrinol. Metab. Clin. N. Am..

[11] Powell-Wiley T.M., Poirier P., Burke L.E., Després J.-P., Gordon-Larsen P., Lavie C.J., Lear S.A., Ndumele C.E., Neeland I.J., Sanders P. (2021). Obesity and Cardiovascular Disease: A Scientific Statement From the American Heart Association. Circulation.

[12] Klein S., Gastaldelli A., Yki-Järvinen H., Scherer P.E. (2022). Why Does Obesity Cause Diabetes?. Cell Metab..

[13] Fantin F., Giani A., Zoico E., Rossi A.P., Mazzali G., Zamboni M. (2019). Weight Loss and Hypertension in Obese Subjects. Nutrients.

[14] Blasco B.V., García-Jiménez J., Bodoano I., Gutiérrez-Rojas L. (2020). Obesity and Depression: Its Prevalence and Influence as a Prognostic Factor: A Systematic Review. Psychiatry Investig..

[15] Kuvat N., Tanriverdi H., Armutcu F. (2020). The Relationship between Obstructive Sleep Apnea Syndrome and Obesity: A New Perspective on the Pathogenesis in Terms of Organ Crosstalk. Clin. Respir. J..

[16] Weihe P., Spielmann J., Kielstein H., Henning-Klusmann J., Weihrauch-Blüher S. (2020). Childhood Obesity and Cancer Risk in Adulthood. Curr. Obes. Rep..

[17] Lega I.C., Lipscombe L.L. (2020). Review: Diabetes, Obesity, and Cancer—Pathophysiology and Clinical Implications. Endocr. Rev..

[18] Butland B., Jebb S., Kopelman P., McPherson K., Thomas S., Mardell J., Parry V. (2007). Tackling Obesities: Future Choices—Project Report.

[19] Locke A.E., Kahali B., Berndt S.I., Justice A.E., Pers T.H., Day F.R., Powell C., Vedantam S., Buchkovich M.L., Yang J. (2015). Genetic Studies of Body Mass Index Yield New Insights for Obesity Biology. Nature.

[20] Loos R.J.F., Yeo G.S.H. (2022). The Genetics of Obesity: From Discovery to Biology. Nat. Rev. Genet..

[21] Robbins L.B., Chang M.-W., Ling J., Brown R. (2022). Psychosocial Factors Affecting the Association between a Healthy Lifestyle Behavior Intervention and Depressive Symptoms in Low-Income Overweight or Obese Mothers with Young Children: A Mediational Analysis. J. Pediatr. Perinatol. Child. Health.

[22] Anekwe C.V., Jarrell A.R., Townsend M.J., Gaudier G.I., Hiserodt J.M., Stanford F.C. (2020). Socioeconomics of Obesity. Curr. Obes. Rep..

[23] Poorolajal J., Sahraei F., Mohamdadi Y., Doosti-Irani A., Moradi L. (2020). Behavioral Factors Influencing Childhood Obesity: A Systematic Review and Meta-Analysis. Obes. Res. Clin. Pract..

[24] WHO Obesity. https://www.who.int/health-topics/obesity.

[25] Ginsburg B.M., Sheer A.J. (2023). Destigmatizing Obesity and Overcoming Inherent Barriers to Obtain Improved Patient Engagement. StatPearls.

[26] Puhl R.M., Himmelstein M.S., Pearl R.L. (2020). Weight Stigma as a Psychosocial Contributor to Obesity. Am. Psychol..

[27] Abiri B., Hosseinpanah F., Banihashem S., Madinehzad S.A., Valizadeh M. (2022). Mental Health and Quality of Life in Different Obesity Phenotypes: A Systematic Review. Health Qual. Life Outcomes.

[28] Rigas G., Williams K., Sumithran P., Brown W.A., Caterson I.D. (2023). Barriers to Progression through Australian Obesity Management Pathways: Survey Data from the ACTION-IO Study. Aust. J. Gen. Pract..

[29] Cuevas A., Alonso R., Contreras Á., Montt D., Rendon A. (2021). Results of the ACTION-IO Survey in Chilean Patients with Obesity and Health Care Providers. Rev. Med. Chile.

[30] Dicker D., Kornboim B., Bachrach R., Shehadeh N., Potesman-Yona S., Segal-Lieberman G. (2020). ACTION-IO as a Platform to Understand Differences in Perceptions, Attitudes, and Behaviors of People with Obesity and Physicians across Countries—The Israeli Experience. Isr. J. Health Policy Res..

[31] Sbraccia P., Busetto L., Santini F., Mancuso M., Nicoziani P., Nicolucci A. (2021). Misperceptions and Barriers to Obesity Management: Italian Data from the ACTION-IO Study. Eat. Weight. Disord..

[32] Iwabu M., Yamauchi T., Shimomura I., Eguchi K., Ogawa Y. (2021). Perceptions, Attitudes and Barriers to Obesity Management: Japanese Data from the ACTION-IO Study. J. Diabetes Investig..

[33] Vázquez-Velázquez V., Laviada-Molina H., García-García E., Sandoval-Diez E., Mancillas-Adame L. (2021). Perceptions, Attitudes, and Barriers to Obesity Care in Mexico: Data From the ACTION-IO Study. Obesity.

[34] Alfadda A.A., Al Qarni A., Alamri K., Ahamed S.S., Abo’ouf S.M., Shams M., Abdelfattah W., Al Shaikh A. (2021). Perceptions, Attitudes, and Barriers toward Obesity Management in Saudi Arabia: Data from the ACTION-IO Study. Saudi J. Gastroenterol..

[35] Lim S., Oh B., Lee S.-H., Kim Y.-H., Ha Y., Kang J.-H. (2020). Perceptions, Attitudes, Behaviors, and Barriers to Effective Obesity Care in South Korea: Results from the ACTION-IO Study. J. Obes. Metab. Syndr..

[36] Salvador J., Vilarrasa N., Poyato F., Rubio M.Á. (2020). Perceptions, Attitudes, and Barriers to Obesity Management in Spain: Results from the Spanish Cohort of the International ACTION-IO Observation Study. J. Clin. Med..

[37] Nawar R., Ibrahim E., Abusnana S., Al Awadi F., Al Hammadi F.H., Farghaly M., Fiad T.M., Aly H., Aly Mohamed Y., Ben Serghin Z. (2021). Understanding the Gaps in Obesity Management in the UAE: Perceptions, Barriers, and Attitudes. Dubai Diabetes Endocrinol. J..

[38] Hughes C.A., Ahern A.L., Kasetty H., McGowan B.M., Parretti H.M., Vincent A., Halford J.C.G. (2021). Changing the Narrative around Obesity in the UK: A Survey of People with Obesity and Healthcare Professionals from the ACTION-IO Study. BMJ Open.

[39] Caterson I.D., Alfadda A.A., Auerbach P., Coutinho W., Cuevas A., Dicker D., Hughes C., Iwabu M., Kang J., Nawar R. (2019). Gaps to Bridge: Misalignment between Perception, Reality and Actions in Obesity. Diabetes Obes. Metab..

[40] Sharma A.M., Bélanger A., Carson V., Krah J., Langlois M.-F., Lawlor D., Lepage S., Liu A., Macklin D.A., MacKay N. (2019). Perceptions of Barriers to Effective Obesity Management in Canada: Results from the ACTION Study. Clin. Obes..

[41] Weir C.B., Jan A. (2023). BMI Classification Percentile And Cut Off Points. StatPearls.

[42] Robinson E. (2017). Overweight but Unseen: A Review of the Underestimation of Weight Status and a Visual Normalization Theory. Obes. Rev..

[43] OECD (2019). The Heavy Burden of Obesity: The Economics of Prevention.

[44] Santomauro D.F., Herrera A.M.M., Shadid J., Zheng P., Ashbaugh C., Pigott D.M., Abbafati C., Adolph C., Amlag J.O., Aravkin A.Y. (2021). Global Prevalence and Burden of Depressive and Anxiety Disorders in 204 Countries and Territories in 2020 Due to the COVID-19 Pandemic. Lancet.

[45] Luppino F.S., de Wit L.M., Bouvy P.F., Stijnen T., Cuijpers P., Penninx B.W.J.H., Zitman F.G. (2010). Overweight, Obesity, and Depression: A Systematic Review and Meta-Analysis of Longitudinal Studies. Arch. Gen. Psychiatry.

[46] French Ministry of Health and Prevention (2019). French National Nutrition Health Program (Plan National Nutrition Santé, PNNS)—2019–2023.

[47] Popoviciu M.-S., Păduraru L., Yahya G., Metwally K., Cavalu S. (2023). Emerging Role of GLP-1 Agonists in Obesity: A Comprehensive Review of Randomised Controlled Trials. Int. J. Mol. Sci..

[48] Oberlin P., Peretti C. (2020). De Bariatric Surgery in France from 1997 to 2018. Surg. Obes. Relat. Dis..

[49] (2023). Haute Autorité de Santé Care Pathway Guide: Overweight and Obesity in Adults. https://www.has-sante.fr/jcms/p_3408871/en/care-pathway-guide-overweight-and-obesity-in-adults.

