# Supplementary material for: ACTION-FRANCE: Insights into Perceptions, Attitudes, and Barriers to Obesity Management in France
Source: J Clin Med. 2024 Jun 15;13(12):3519. doi: 10.3390/jcm13123519 (PMC11204730; doi:10.3390/jcm13123519)
Supplement: Supplementary file 1 [file jcm-13-03519-s001.zip › jcm-2990223-supplementary.pdf]

Supplementary Data

ACTION-FRANCE: Insights into Perceptions, Attitudes, and Barriers to Obesity Management in France

Laurence Salle[1,2 *], Olivier Foulatier[3,4], Muriel Coupaye[5,6] Vincent Frering[7], Alina Constantin[3], Anne-Sophie Joly[8], Ben Braithwaite[9], Fella Gharbi[10], Lysiane Jubin[10]

Table S1 - Stratifications of respondents

Stratifications	Criteria
**People with obesity (PwO)**	
Current weight loss success (S) or non-success (NS)	• Success (S): I have generally lost weight over the past year.<br>• Non-success (NS): Either gained weight year or weight has remained stable over the past.
Obesity class, based on self-reported weight and height	• Overweight: body mass index (BMI) 27-29,9 kg/m^2<br>• Class I: body mass index (BMI), 30-34.9 kg/m^2<br>• Class II: BMI, 35-39.9 kg/m^2<br>• Class III: BMI, ≥40 kg/m^2
Treatment-seeking behavior	• Actively seeking treatment: has spoken to a healthcare provider (HCP) about a weight-loss plan within the previous 6 months.<br>• Non-treatment seeking: has not spoken to a healthcare provider (HCP) about a weight-loss plan within the previous 6 months
**Healthcare professionals (HCPs)**	
Type	• Doctors<br>• Dieticians<br>• Other professions (nurses, pharmacists, psychologist, physiotherapist)
Obesity specialists (OS), or non-obesity specialists (NOS) *	• OS: ≥50% of adult patients seen primarily for weight management<br>• NOS: <50% of adult patients seen primarily for weight management

* only physicians

Table S2 - Patient's Comorbidities

Comorbidities	BMI<30 kg/m², N = 183	Class I, N = 529	Class II, N = 276	Class III, N = 238	Overall, N = 1,226
**Hypertension, n (%)**	47 (25.7)	173 (32.7)	91 (33.0)	92 (38.7)	403 (32.9)
**Cardiovascular diseases, n (%)**	18 (9.8)	41 (7.8)	29 (10.5)	26 (10.9)	114 (9.3)
**Depression/Anxiety, n (%)**	56 (30.6)	152 (28.7)	116 (42.0)	100 (42.0)	424 (34.6)
**Dyslipidemia, n (%)**	17 (9.3)	68 (12.9)	37 (13.4)	35 (14.7)	157 (12.8)
**Infertility, n (%)**	5 (2.7)	14 (2.6)	7 (2.5)	13 (5.5)	39 (3.2)
**Liver disease, n (%)**	5 (2.7)	33 (6.2)	25 (9.1)	28 (11.8)	91 (7.4)
**Obstructive sleep apnea, n (%)**	15 (8.2)	74 (14.0)	59 (21.4)	84 (35.3)	232 (18.9)
**Osteoarthritis, n (%)**	4 (2.2)	12 (2.3)	6 (2.2)	8 (3.4)	30 (2.4)
**Metabolic syndrome, n (%)**	2 (1.1)	5 (0.9)	12 (4.3)	12 (5.0)	31 (2.5)
**Stomach or intestinal problems, n (%)**	13 (7.1)	64 (12.1)	42 (15.2)	26 (10.9)	145 (11.8)
**Pre-diabetes, n (%)**	4 (2.2)	21 (4.0)	21 (7.6)	22 (9.2)	68 (5.5)
**Diabetes (type II), n (%)**	27 (14.8)	48 (9.1)	41 (14.9)	39 (16.4)	155 (12.6)
**Diabetes (type I), n (%)**	7 (3.8)	17 (3.2)	13 (4.7)	11 (4.6)	48 (3.9)
**Cancer, n (%)**	8 (4.4)	26 (4.9)	11 (4.0)	11 (4.6)	56 (4.6)
**Polycystic ovary syndrome (PCOS), n (%)**	3 (1.6)	24 (4.5)	15 (5.4)	18 (7.6)	60 (4.9)
**Eating disorder, n (%)**	18 (9.8)	42 (7.9)	54 (19.6)	47 (19.7)	161 (13.1)
**Urinary or fecal incontinence, n (%)**	3 (1.6)	24 (4.5)	15 (5.4)	22 (9.2)	64 (5.2)
**Other endocrinopathies, n (%)**	7 (3.8)	50 (9.5)	37 (13.4)	33 (13.9)	127 (10.4)
**Dental problems, n (%)**	20 (10.9)	81 (15.3)	39 (14.1)	35 (14.7)	175 (14.3)
**None of these pathologies, n (%)**	48 (26.2)	125 (23.6)	47 (17.0)	27 (11.3)	247 (20.1)
**Other pathology, n (%)**	12 (6.6)	30 (5.7)	19 (6.9)	19 (8.0)	80 (6.5)

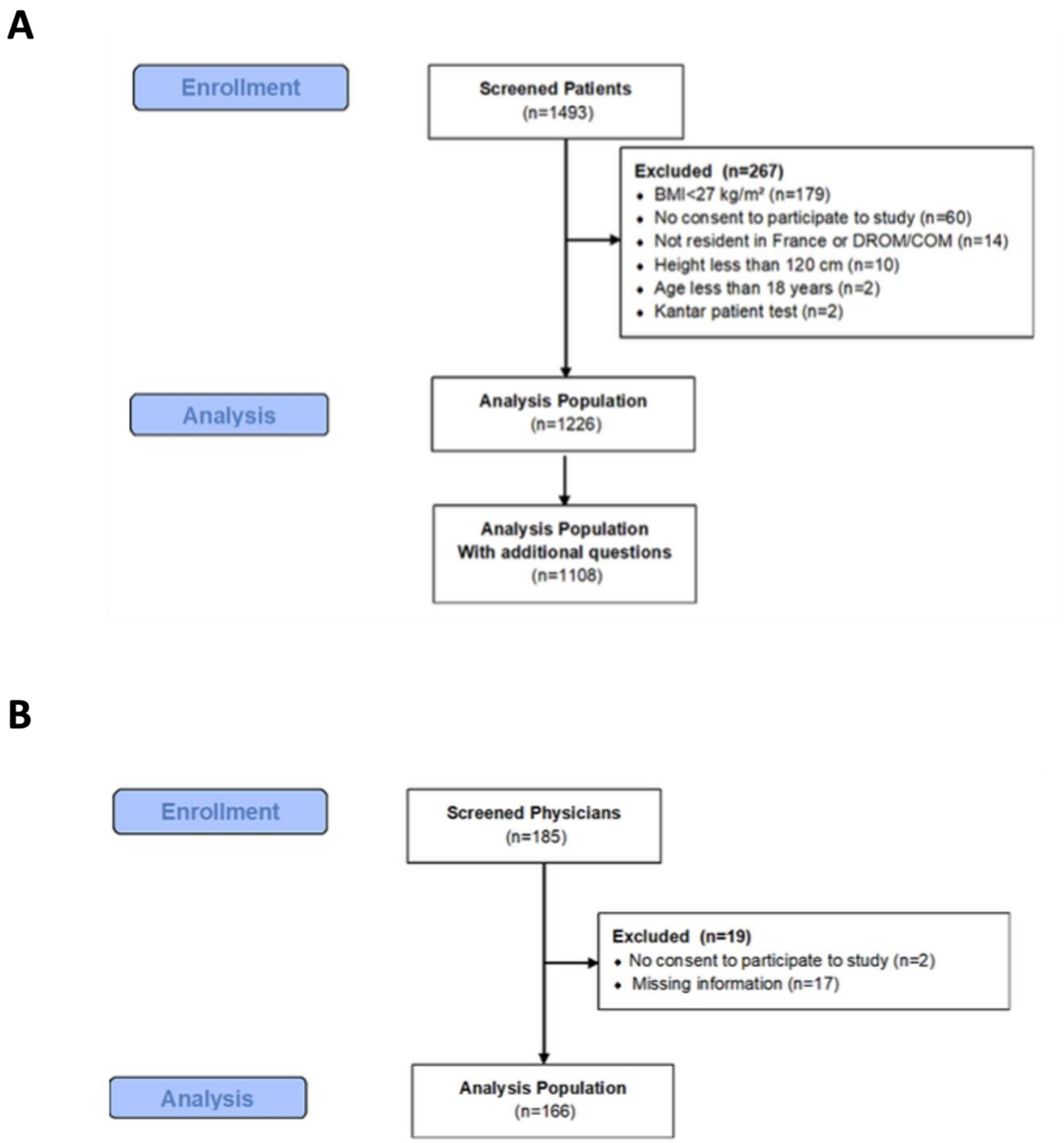

Figure S1: Flow chart of the Action-FRANCE study for PwO (A) and HCPs (B).

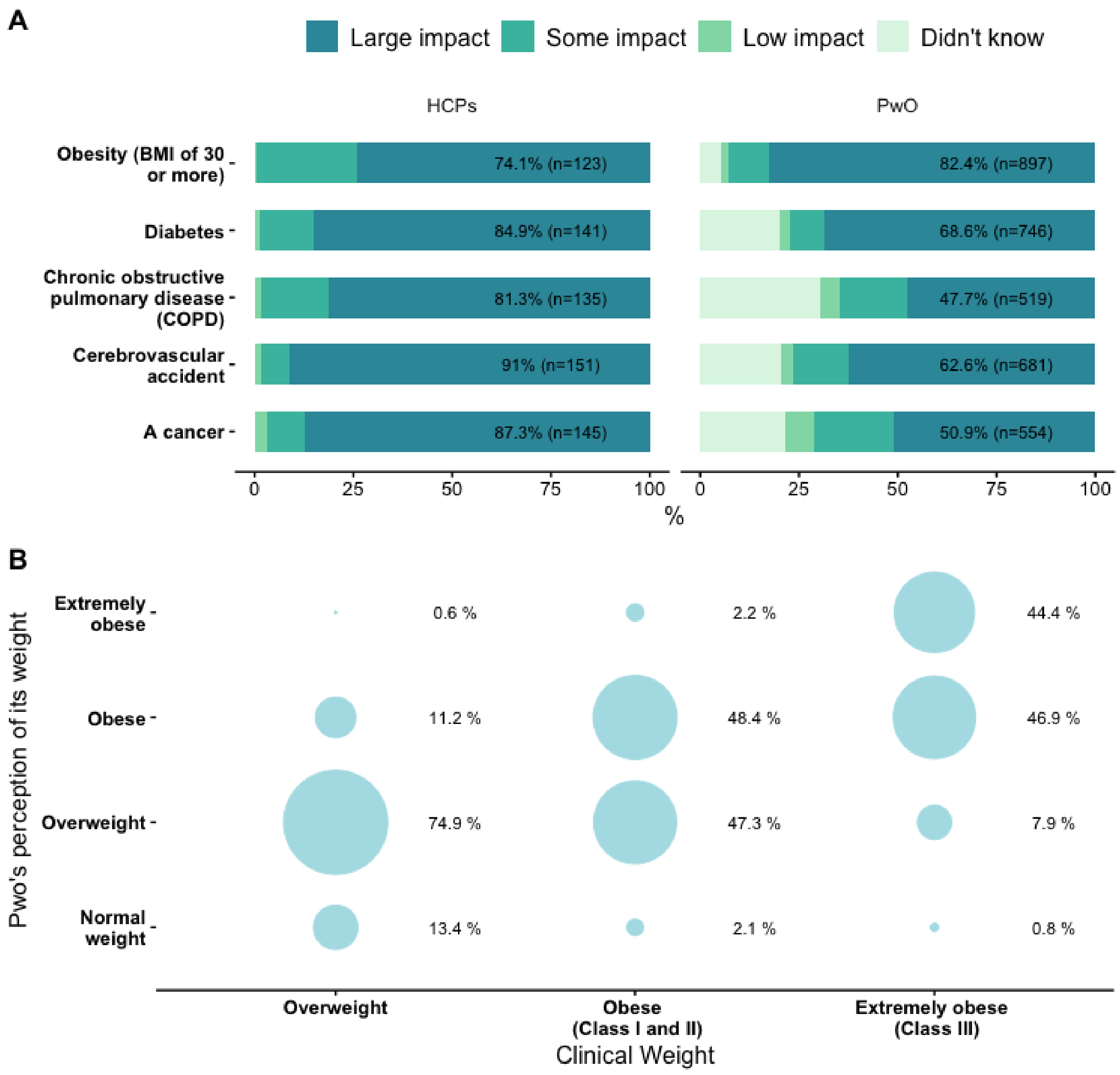

Figure S2: Perception of obesity and impact of obesity on general health status by HCPs and PwO. (A) Response to the question, 'What is the influence of each item on an individual's health' according to both population. The percentage represents the proportion of responses for each Likert scale item. (B) PwOs' perception of their weight in relation to their clinical weight. Clinical weight was defined as follows : Overweight (BMI <= 29.9), Obese (BMI >= 30 and BMI <= 39.9, Class I and II) et Extremely Obese (BMI >= 40, Class III). The percentage corresponds to the total number of respondents by clinical category.
